# Disruptions in Resting State Functional Connectivity and Cerebral Blood Flow in Mild Traumatic Brain Injury Patients

**DOI:** 10.1371/journal.pone.0134019

**Published:** 2015-08-04

**Authors:** Chandler Sours, Jiachen Zhuo, Steven Roys, Kathirkamanthan Shanmuganathan, Rao P. Gullapalli

**Affiliations:** 1 Magnetic Resonance Research Center, University of Maryland School of Medicine, Baltimore, Maryland, United States of America; 2 Department of Diagnostic Radiology & Nuclear Medicine, University of Maryland School of Medicine, Baltimore, Maryland, United States of America; University of California, San Francisco, UNITED STATES

## Abstract

Mild traumatic brain injury (mTBI) is often occult to conventional imaging techniques. However, there is growing evidence that mTBI patients who lack evidence of structural intracranial injury may develop post-concussive syndrome (PCS). We investigated longitudinal alterations in resting state functional connectivity (rs-FC) in brain networks in a population of 28 patients compared to 28 matched control participants. Rs-FC and cerebral blood flow (CBF) within the nodes of the Default Mode Network (DMN) and Task Positive Network (TPN) were assessed at three time points including acute, sub-acute, and chronic stages following mTBI. Participants received the Automated Neuropsychological Assessment Metrics (ANAM) to assess cognitive performance. Main findings indicate that despite normalized cognitive performance, chronic mTBI patients demonstrate increased rs-FC between the DMN and regions associated with the salience network (SN) and TPN compared to the control populations, as well as reduced strength of rs-FC within the DMN at the acute stage of injury. In addition, chronic mTBI patients demonstrate an imbalance in the ratio of CBF between nodes of the DMN and TPN. Furthermore, preliminary exploratory analysis suggests that compared to those without chronic PCS, patients with chronic PCS reveal an imbalance in the ratio of CBF between the DMN nodes and TPN nodes across multiple stages of recovery. Findings suggest that the altered network perfusion with the associated changes in rs-FC may be a possible predictor of which mTBI patients will develop chronic PCS.

## Introduction

Traumatic brain injury (TBI) is one of the most prevalent neurological disorders in the United States, resulting in a large emotional burden on patients and a vast financial burden on the nation. The Center for Disease Control (CDC) estimates that there are 1.7 million cases of TBI each year [1]. Although greater than 75% of these injuries are considered mild, many of these patients have poor long-term outcomes[2]. One of the most common types of damage induced by TBI is diffuse axonal injury (DAI), which is commonly missed by conventional CT and MR imaging especially in mild cases [3,4]. This makes it challenging to predict which of these individuals will go on to suffer from persistent symptoms following the initial injury [5], a condition known as post-concussive syndrome (PCS) [6,7].

Therefore, further investigation of advanced neuroimaging techniques to better characterize mild TBI (mTBI) is needed. One possible technique is resting state functional MRI, which can be used to characterize the interactions both within and between various neural networks. This interaction is calculated by determining functional connectivity, which can be defined as “the temporal correlation of a neurophysiological index measured in different brain areas” [8]. Two common resting state networks are the Default Mode Network (DMN) and the Task Positive Network (TPN), also referred to as the executive network. The DMN is a network of regions that is consistently deactivated, or “turned off” during task-related activities but demonstrates increased activity during resting conditions [9]. The DMN represents internally directed, self-reflective processes [10] and includes the posterior cingulate cortex (PCC), lateral parietal cortex (LPC), anterior cingulate cortex (ACC), and medial prefrontal cortex (MPFC) [11,12]. In contrast, the TPN is recruited during externally directed behavior and includes the bilateral dorsolateral prefrontal cortex (DLPFC) and posterior parietal cortex [13].

In ideal conditions, it has been shown that these two networks are anti-correlated at rest, which is represented by a negative resting state functional connectivity (rs-FC) [12]. It has been further suggested that the failure to adequately suppress activity of the DMN during cognitively demanding tasks results in a state of mind wandering or inattention, therefore, hindering task performance[14]. Termed the Default Mode Interference Hypothesis, support for this theory results from studies in healthy volunteers where momentary lapses in attention were associated with a reduction in task induced deactivations of the DMN [15]. Furthermore, this balance between activation of the TPN and deactivation of the DMN during a task has been shown to be disrupted in multiple conditions with cognitive deficits similar to those of mTBI, including healthy aging [16,17], sleep deprivation [18], schizophrenia [19], attention deficit and hyperactivity disorder [20], post traumatic stress disorder (PTSD) [21], and sub-acute mTBI [22]. Additional data suggest that the modulation of the TPN and DMN is mediated by a third network, the Salience Network (SN) [23–25]. Given these findings, we propose that this imbalance in internally versus externally directed mental processes present during resting conditions may contribute to long-term PCS experienced by a subset of mTBI patients.

TBI has been shown to reduce rs-FC in multiple networks including interhemispheric rs-FC [26–28], the motor network [29], the TPN [30–32], and the DMN [30,33–37]. In addition, recent data suggests that there is increased rs-FC between the DMN and TPN in mTBI populations [30] and specifically in mTBI patients with self-reported memory complaints [38]. However, it is not clear whether in vivo changes in resting state conditions may be the result of altered neurovascular coupling, altered perfusion patterns as a result of structural damage induced by DAI, or are truly the result of changes in neural activity [39]. In TBI, these vascular changes may be induced by the breakdown of the blood brain barrier known to occur after injury [40]. Furthermore, the secondary injury cascade associated with TBI including reduced energy and glucose metabolism, increased inflammatory response, increased oxidative stress [40], and the diffuse reactive astrogliosis [41] may all be the result of an altered regional brain perfusion.

Using SPECT imaging, it has been shown that symptomatic mTBI patients demonstrate reduced regional CBF in the frontal, prefrontal, and temporal cortices in the chronic stage of injury (greater than two years post injury) [42]. While CT perfusion, SPECT imaging, and dynamic susceptibility contrast perfusion imaging [43] have been used in TBI research for years, the arterial spin labeling (ASL) technique to measure brain perfusion has only recently been applied to TBI in both human populations and animal models. ASL is a non-invasive technique that uses protons in the arterial blood as an endogenous contrast agent eliminating the need for an exogenous contrast agent such as gadolinium. In a rodent lateral fluid percussion model of TBI, Hayward and colleagues noted increased regional CBF in the thalamus at the chronic stage that was positively correlated with increased vessel density in this region [44]. This increased vessel density within the thalamus was associated with reduced performance on the Morris water maze, suggesting that altered regional perfusion influences chronic cognitive performance. Furthermore, severe TBI patients show a global hypoperfusion at rest including regions of the posterior cingulate cortex (PCC), thalamus and disperse frontal regions [45]. In contrast to the animal studies [44], mTBI patients demonstrate reduced resting CBF in the thalamus, which was associated with reduced performance on measures of neurocognitive functioning [45]. Given the above findings, we hypothesize that information from resting state CBF changes in the mTBI population can potentially supplement the interpretation of information obtained from the rs-FC analyses. While regional alterations in perfusion are noted following mTBI, modifications in the perfusion of specific networks are rarely assessed following trauma. Evidence exists demonstrating that during resting conditions, perfusion within the DMN is significantly higher than perfusion within the TPN among normal populations [46]. To our knowledge this balance of resting CBF within the TPN and DMN has not been investigated in an mTBI population in the context of the DMN Interference Hypothesis. Understanding the CBF changes in the context of changes in the network connectivity may provide further insights into the pathophysiology of mTBI.

In this study we followed a group of mTBI patients across the acute, sub-acute, and chronic stages of injury in order to investigate longitudinal changes in rs-FC within and between the DMN and TPN compared to control participants. In addition, we assessed whether mTBI patients exhibit alterations in the balance of resting perfusion within the DMN and TPN compared to control participants across the first 6 months following injury.

## Materials and Methods

### Ethics Statement

This study was approved by the International Review Board at the University of Maryland and all participants provided written informed consent and HIPAA compliance.

### Participants

MTBI patients were prospectively recruited between March 2010 and May 2012 from the R Adam Cowley Shock Trauma Center at the University of Maryland Medical Center as part of a larger imaging protocol using a combination of advanced MR imaging and neuropsychological assessments including the Automated Neuropsychological Assessment Metrics (ANAM) [47]. All participants were over the age of 18. Patients were screened and excluded for history of neurological and psychiatric illness, history of stroke, history of brain tumors or seizures, and contraindications to MR. Only those patients with an admission Glasgow Coma Score (GCS) of 13–15, mechanism of injury consistent with trauma, and either (1) a positive head CT or (2) altered mental status and/or loss of consciousness (LOC) were included in this study. While questions regarding LOC and altered mental status were included in our interview process, participants were generally unable to answer questions regarding duration of these events or gave variable responses during different visits leading us to decide that the length of LOC or altered mental status given to us by the participants was an unreliable measure in our patient population. However, based on the information collected from the first responders, 18/28 patients had LOC, 3/28 patients did not have LOC. Data regarding LOC for the remaining 7 patients was unavailable due to the fact that no other individuals were present during the accident. However, of these 7 patients, they either had a positive head CT (n = 1) or noted altered mental status (n = 6) when the first responders arrived.

For the analysis presented in this manuscript, only participants who completed longitudinal resting state fMRI across the three time points of interest were included resulting in a final patient population of 28 mTBI patients (38.9+/-15.9yrs, 18M:10F). Individual patient clinical characteristics, mechanisms of injury, and CT and MRI interpretation by board certified trauma radiologist (K.S.) are shown in S1 Table. Presence of intracranial injury on MRI was determined by the examination of conventional MR images (T1, T2, FLAIR, and SWI). Five out of the 28 patients had either CT abnormalities while one additional patient had evidence of MRI abnormalities not noted on the convention CT, resulting in 6 mTBI patients with noted intracranial injury. Twenty-eight neurologically intact participants (39.25+/-17.2yrs, 16M:12F) that were age and education matched served as the control population. Participant demographic information is shown in Table 1.

**Table 1:** Participant Demographics.

	N	Age	Sex	Education	Days Acute	Days Sub-Acute	Days Chronic	+CT	+MR
**Control**	**28**	39.3 ± 17.2	16M: 12F	14.7 ± 2.2	NA	NA	NA	NA	NA
**mTBI**	**28**	38.9 ± 15.9	18M: 10F	13.9 ± 2.7	6 ± 3	36 ± 13	198 ± 26	5/28	6/28
**PCS**	**12**	46.3 ± 14.1*	6M: 6F	13.3 ± 2.1	7 ±3	35 ± 8	196 ± 23	2/12	3/12
**No PCS**	**16**	33.3 ± 15.3*	12M: 4F	14.3 ± 3.0	6 ± 3	36 ± 15	199 ± 29	3/16	3/16

* p <0.05 based on independent t-tests

All 28 mTBI patients received rs-fMRI at three time points. An initial stage (referred to as acute stage) within 11 days (average 6 +/-3 days, range 1–11 days), a sub-acute stage approximately 1 month post injury (average 36 +/-13 days, range 25–88 days), and a chronic stage (average 198 +/-26 days, range 137–266 days). At each time point the patients also received a resting state perfusion scan, using the pulsed arterial spin labeling technique (PASL). However, due to motion artifacts (translation greater than 3mm or rotation greater than 3 degrees), resting state data from 1 patient at the acute stage was excluded from analysis, and PASL data from 2 patients at the acute stage and 2 patients at the sub-acute stage were excluded from analysis. The 28 control participants received rs-fMRI and PASL scans at a single time point.

In an exploratory analysis, mTBI patients were further subdivided into two cohorts of those with and without PCS at the chronic stage based on self-reported symptoms on the Modified Rivermead Post-Concussion Symptoms Questionnaire (RPQ) [48]. The RPQ asks participants to rate a series of common symptoms following TBI on a scale of 0–4. Based on the International Classification of Disease tenth revision (ICD10) symptom criteria for PCS we defined PCS as reporting 3 or more of the following symptoms lasting for greater than three months: headaches, dizziness, sleep disturbances, trouble concentrating, fatigue, memory problems, and irritability at the chronic stage [49]. Twelve of the mTBI patients qualified as having PCS at 6 months while 16 did not qualify as having PCS.

Resting state fMRI data from a subset of this patient population has previously been published investigating the association between imaging findings and memory complaints (n = 16 at the sub-acute stage) [38] as well the associations between interhemispheric functional connectivity and cognitive performance (n = 18 at the acute and sub-acute stage of injury) [28].

### Neuropsychological Assessment

Patients underwent neuropsychological assessment at all three visits. However, due to the clinical condition or presence severe of post-concussive symptoms at time of the MRI, only 19 of the 28 mTBI patients at the acute stage and 27 out of the 28 mTBI patients at the sub-acute stage completed the neuropsychological assessment. One control participant declined participation in the neuropsychological assessment resulting a control population of 27 participants for the neuropsychological assessment. Imaging data was included in the analysis for this control participant. Due to the possible selection bias caused by one third of the mTBI patients lacking neuropsychological testing at the initial stage, we have excluded analysis of behavioral data for the acute time point. The level of cognitive functioning was assessed by the administration of the Mini Mental State Exam (MMSE) and Military Acute Concussion Evaluation (MACE) [50] at each visit. Global life satisfaction was assessed using Satisfaction with Life Scale (SWLS) [51]. Patient outcome was assessed by the Glasgow Outcome Scale Extended (GOSE) [52] and Disability Rating Scale (DRS) [53] at 6 months.

The ANAM battery consists of seven subtests assessing processing speed, memory, and attention. The specific subtests included in this battery are the code substitution (CS), code substitution delayed (CSD), match to sample (MTS), math processing (MATH), procedural reaction time (PRT), simple reaction time (SRT), simple reaction time repeat (SRT2) [54]. From each subtest, an individual throughput score is calculated which is a single measure encompassing both accuracy and reaction time. Specifically, the throughput score is the number of correct responses per total amount of time a participant took to respond for each trial, expressed as the number of correct responses per minute [55]. We opted to examine a weighted throughput score (WT-TH), which has previously been referred to as an Index of Cognitive Efficiency [56]. The WT-TH was determined as an overall measure of performance on the ANAM and is given as WT-TH = (CS*4.35+CSD*5+MTS*6.63+MATH*8.37+PRT*2.18+SRT+SRT2)/7.

### MR Data Acquisition

All imaging was performed on a Siemens Tim-Trio 3T MRI scanner using a 12 channel receive only head coil. A high resolution T1-MPRAGE (TE = 3.44 ms, TR = 2250 ms, TI = 900 ms, flip angle = 9°, resolution = 256 × 256 × 96, FOV = 22 cm, sl. thick. = 1.5 mm) was acquired for anatomic reference with slices parallel to the anterior and posterior commissure points (AC-PC). For the rs-fMRI scan, T2*-weighted images were acquired using a single-shot EPI sequence (TE = 30 ms, TR = 2000 ms, FOV = 230 mm, resolution = 64 × 64) with 36 axial slices (sl. thick. = 4 mm) over 5 min 42 seconds that yielded 171 volumes. The perfusion scan used the pulsed arterial spin labeling (PASL) technique based on single-shot EPI readout (TE = 11ms, TR = 2500ms, FOV = 230mm, resolution = 64×64) with 16 slices (sl. thick. = 5mm with 1mm gap) to cover the central portion of the brain with the spatial location of these slices matching the structural images. Forty-five pairs of labeled and control volumes were acquired following the initial acquisition of the constant equilibrium magnetization (M0) volume. The PASL imaging data was taken over 3 min and 57 seconds. During the resting state scans, participants were instructed to rest peacefully with eyes closed.

### Data Analysis

#### Resting State Data Preprocessing

Preprocessing of the imaging data was performed using SPM 8 (http://www.fil.ion.ucl.ac.uk/spm) and included motion correction of the time series, slice timing correction, band pass filtering (.009Hz < f < .08Hz), and registration of all the 171 volumes to the first volume of the time series. Both the T1-MPRAGE and resting state series were directly spatially normalized to standard space using the Montreal Neurological Institute (MNI) templates available in SPM 8. The T1 template was used for normalization of the T1-MPRAGE and the EPI template was used for the normalization of the resting state time series. The SPM normalization step uses the normalized mutual information to calculate the optimum 12-parameter affine transformation (3 translations, 3 rigid-body rotations, 3 shears, and 3 zooms) to match the size and position of the images. The resting state series was resampled to a spatial resolution of 2.0 mm isotropic. Spatial smoothing was then applied to the resting state data using a 5 mm Gaussian kernel. Individual T1-MPRAGE images in MNI space were segmented into white matter (WM), gray matter (GM) and cerebral spinal fluid (CSF). The segmented masks thus created were used to account for time series variance from the non-neuronal contributions of CSF and WM. The mean BOLD time series from the WM mask, CSF mask, and the 6 motion correction parameters were included in the model as regressors to remove the variance related to non-neuronal contributions and motion.

#### Resting State Voxel-based Analysis

The CONN-fMRI Functional Connectivity toolbox v13.h (http://www.nitrc.org/projects/conn) was used to process the resting state data and create average group networks for the DMN and the TPN. For the DMN, the reference time series was selected from a 5mm spherical region of interest (ROI) in the posterior cingulate cortex (PCC) centered at (-5,-53,41) on the MNI template based on [12]. For the TPN, we selected two reference time series from bilateral 5mm spheres in the DLPFC centered at (-42,34,20) and (44,36,20) [12]. The mean BOLD time series for the above seed ROIs were extracted and correlated with the time series of each voxel within the entire brain. For the TPN, the time series from the right and left DLPFC were averaged before correlating with each voxel’s time series from the entire brain. Correlations were converted to normalized z-scores within the CONN-fMRI functional connectivity toolbox prior to further analysis.

Within group rs-FC maps of the DMN and TPN were created using SPM8. Positive functional connectivity maps were thresholded at a voxel wise *p*-value of 0.001 (uncorrected) and cluster extent threshold of *p*-value of 0.001 using a family wise error correction for multiple comparisons. Voxel wise functional connectivity maps were made separately for the control group, and the mTBI groups at each of the three time points. All between group contrast maps were created and were thresholded at voxel wise *p*-value of 0.005 (uncorrected) and cluster extent threshold of *p*-value of 0.05 using a family wise error correction for multiple comparisons.

#### Resting State ROI-based Analysis

**Fig 1:**
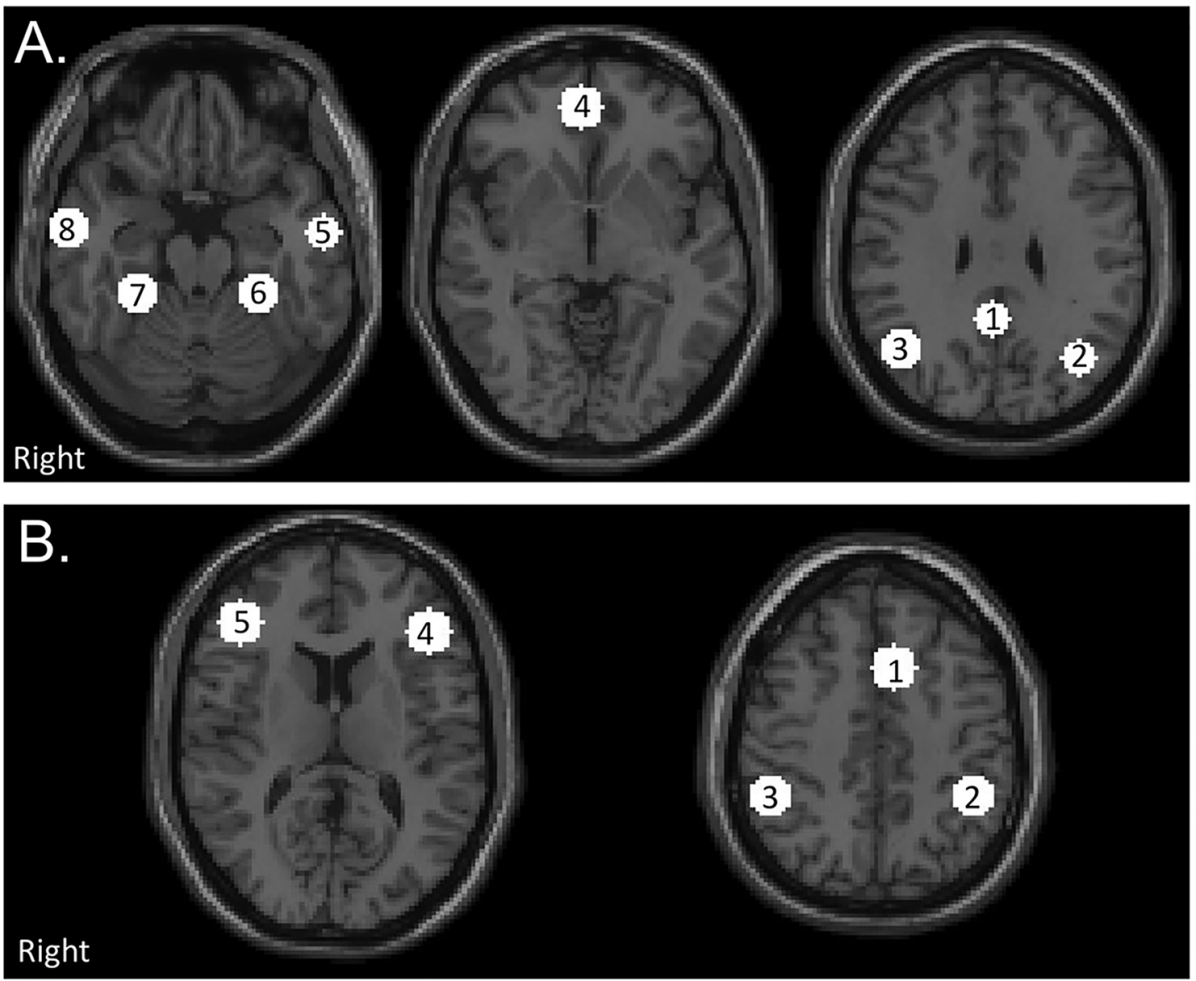
Location of network regions of interests (ROIs). (A) Default Mode Network (DMN). 1) PCC Posterior cingulate cortex (PCC) 2) Left Lateral Parietal (LLP) 3) Right Lateral Parietal (RLP) 4) Medial Prefrontal Cortex (MPFC) 5) Left Inferior Temporal Gyrus (L ITG) 6) Right Inferior Temporal Gyrus (R ITG) 7) Left Medial Temporal Lobe (L MTL) 8) Right Medial Temporal Lobe (R MTL). (B) Task Positive Network (TPN). 1) Premotor Area (PM) 2) Left Supramarginal Gyrus (L SMG) 3) Right Supramarginal Gyrus (R SMG) 4) Left Dorsolateral Prefrontal Cortex (L DLPFC) 5) Right Dosolateral Prefrontal Cortex (R DLPFC). Networks consists of 10 mm spherical ROIs centered around the peak correlated voxel with the PCC for the DMN and the bilateral DLPFC for the TPN from the results of an independent control group thresholded at a voxel wise *p*-value of 0.001 (uncorrected) and cluster extent threshold of *p*-value of 0.001 (FWE).

ROIs were created using a 10mm sphere centered at the peak voxel of each significantly correlated cluster for the DMN and TPN based the voxel wise group connectivity maps of the of an independent control group [57] created using identical ROIs to the voxel-based analysis performed in this present analysis. The DMN and TPN ROIs are shown in Fig 1. Coordinates of ROIs are presented in S2 Table. For each participant, the average time series was extracted from each ROI and pairwise correlations matrices were created. Average correlations matrices were created for the control group, and the mTBI group at each visit (acute, sub-acute, and chronic). Within network connectivity was determined by calculating the average of the pairwise connectivity measures for the 8 DMN ROIs (DMN rs-FC) and the average of the pairwise connectivity measures for the 5 TPN ROIs (TPN rs-FC). Between network connectivity was determined by calculating the average of the pairwise connectivity measure between the 8 DMN ROIs and the 5 TPN ROIs (DMN-TPN rs-FC).

#### PASL Preprocessing

CBF maps were generated using in-house MATLAB (Mathworks Inc, Natick, MA) program as in [58]. Briefly, a Gaussian blur (6mm FWHM) was first applied to the images. After spatial smoothing, all images were motion corrected to the first M0 image through a 6-DOF image registration using AFNI. The M0, control, and labeled images were then extracted from the motion corrected series, and the labeled images were time-shifted to match the control images. The motion corrected control and time-shifted labeled images were then pair-wise subtracted and averaged to produce the mean perfusion-weighted image. CBF maps were estimated from the mean perfusion-weighted image and the M0 image based on formulas provided in [58] (units of mL/100g/min). Next, the T1-MPRAGE in native space was normalized to MNI space using the SPM normalization process using the 12-parameter affine transformation. The T1-MPRAGE in native space was coregistered to the ASL data in native space using the SPM coregistration process using a 12-parameter affine transformation. The DMN and TPN ROIs generated from the resting state analysis were transformed from MNI space to native ASL space via a two-step process. First the MNI space ROIs were transformed to native space and registered to the T1-MPRAGE using the inverse of the transformation matrix from the T1-MPRAGE to MNI space normalization step. Second the transformation from the coregistration of the T1-MPRAGE to the ASL space was applied to the ROIs, effectively registering the MNI space ROIs to native ASL space for subsequent analysis. All registrations were visually assessed to ensure proper registration. A gray matter (GM) mask from segmentation of the T1-MPRAGE was used to mask each ROI from which the GM CBF values were obtained for each ROI. Average CBF values for the DMN and TPN as well as a CBF ratio (TPN CBF/DMN CBF) were calculated.

### Statistical Analysis

Differences in demographic characteristics between the control group and mTBI group were determined using unpaired t-tests. Group differences in measures of rs-FC (DMN rs-FC, TPN rs-FC, and DMN-TPN rs-FC) and rs-CBF (DMN CBF, TPN CBF, and CBF ratio) between the control group and mTBI group were tested using analysis of covariance (ANCOVAs) considering age as a covariate at each time point (acute, sub-acute, and chronic) separately. Longitudinal changes in imaging measures (rs-FC and rs-CBF measures) within the first 6 months following mTBI were determined using repeated measures analysis of covariance (ANCOVAs) considering age as a covariate with visit as the within group variable (acute, sub-acute, or chronic) implemented in SPSS. Longitudinal changes in neuropsychological measures within the first 6 months following mTBI were determined using repeated measures ANCOVAs considering age as a covariate with visit as the within group variable (sub-acute or chronic) implemented in SPSS. Within group differences between DMN CBF and TPN CBF were calculated using paired t-tests. Results shown are uncorrected for multiple comparisons.

## Results

### Participants

Table 1 summarizes the demographic information for the study and control groups. There were no differences between the mTBI group and control group in age (p = 0.98), sex (p = 0.59), or years of education (p = 0.19). There were no differences between the mTBI group with and without chronic PCS in sex (p = 0.18), education (p = 0.31), and days post injury (acute: p = 0.50; sub-acute: p = 0.83; and chronic p = 0.75). In addition, the two groups had similar percentages of CT or MR positive patients (19% CT/MR positive in no PCS group vs 25% CT/MR positive in PCS group). The PCS group was however older than the group without PCS (p = 0.030)

### Neuropsychological Assessment

#### Cross Sectional Analysis

Both the control group and mTBI group at sub-acute and chronic time points performed similarly on the MACE (sub-acute: p = 0.73; chronic: p = 0.76). There were no differences in ANAM performance as measured by the WT-TH between the control group and mTBI group at either time point (sub-acute: p = 0.40; chronic: p = 0.46). There were no differences in global life satisfaction as measured with the SWLS between the mTBI and control group at the sub-acute (p = 0.352) or chronic (p = 0.453) stages of injury.

#### MTBI Longitudinal Analysis

There were no longitudinal differences between the sub-acute and chronic time points in the mTBI group for the MACE (F = 0.003; p = 0.956), ANAM performance (F = 0.496; p = 0.500), or SWLS (F = 0.087; p = 0.770).

### Resting State fMRI-Voxel Based Analysis

#### Cross Sectional Analysis

**Fig 2:**
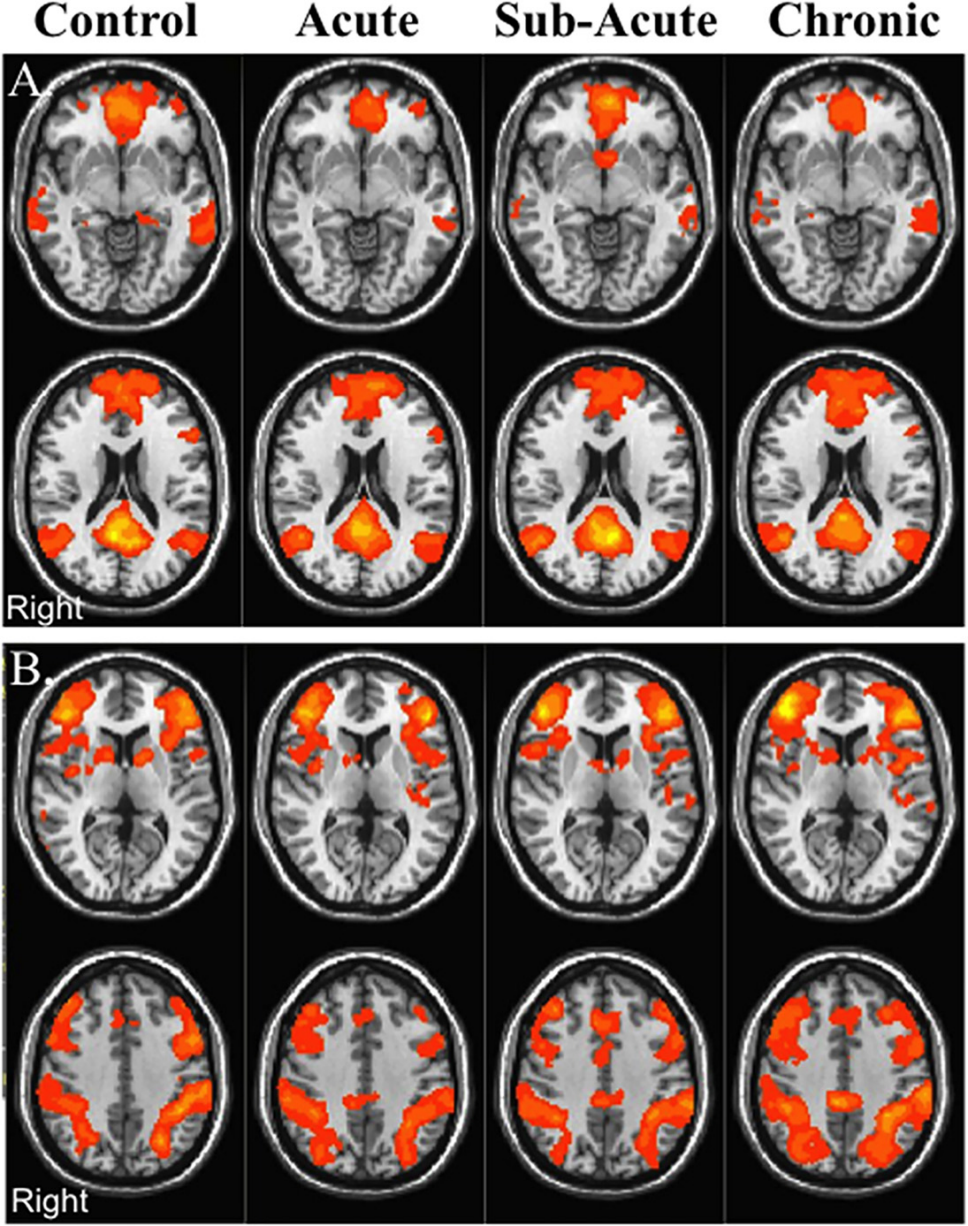
Within group rs-FC maps for control participants and mTBI patients. (A) Default Mode Network (DMN). (B) Task Positive Network (TPN). Maps are thresholded at a voxel wise *p*-value of 0.001 (uncorrected) and cluster extent threshold of *p*-value of 0.001 using a family wise error correction for multiple comparisons.

**Fig 3:**
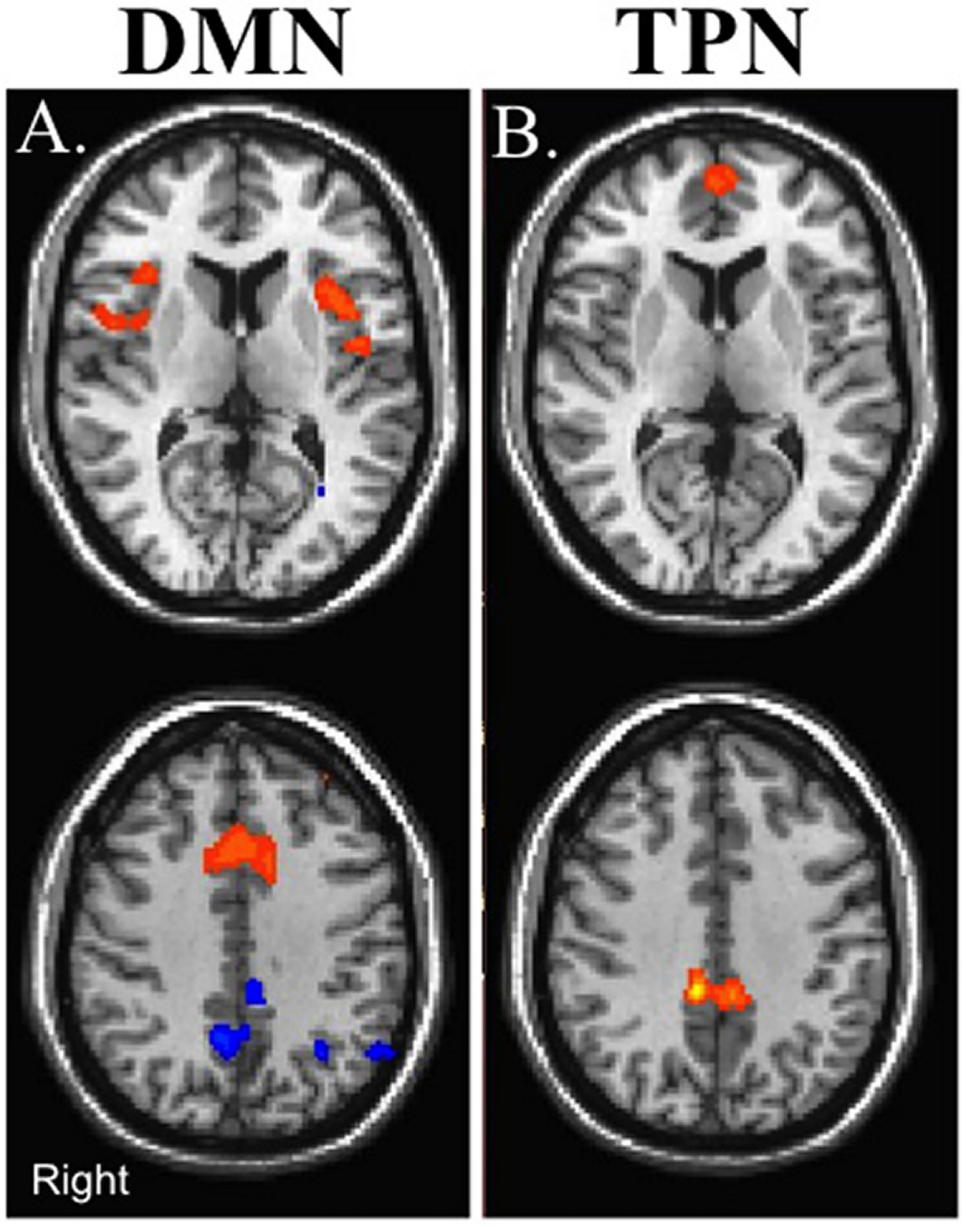
Between group contrast maps for control participants versus mTBI group in the chronic stage. (A) DMN (B) TPN. Warm colors represent regions of increased rs-FC in the mTBI group and cool colors represent regions of reduced rs-FC in the mTBI group. Maps are thresholded at voxel wise *p*-value of 0.005 (uncorrected) and cluster extent threshold of *p*-value of 0.05 using a family wise error correction for multiple comparisons.

Fig 2 represents the within group voxel based results for the DMN and the TPN for the control group and the mTBI group across the three visits (Fig 2). Based on threshold of voxel wise *p*-value of 0.005 (uncorrected) and cluster extent threshold of *p*-value of 0.05 FWE corrected, mTBI patients did not demonstrate any differences in rs-FC with the DMN compared to the control group in the acute or sub-acute stages. However, in the chronic stage, mTBI patients revealed increased rs-FC between the PCC node of the DMN with regions in the TPN such the dorsal anterior cingulate cortex (dACC)/premotor area (PMA), bilateral insular cortex (In), and L DLPFC (Fig 3A; Table 2), suggesting increased rs-FC between the DMN and TPN. In addition, compared to the control group, mTBI patients demonstrated reduced rs-FC between the PCC node of the DMN and other nodes of the DMN including the left supramarginal gyrus (L SMG) and dorsal posterior cingulate cortex (dPCC) in the chronic stage (Fig 3A; Table 2), suggesting reduced within DMN rs-FC. Based on voxel wise analysis, mTBI patients did not demonstrate altered rs-FC with the TPN at the acute or sub-acute stages compared to the control group. However, at the chronic stage, mTBI patients had increased rs-FC with the TPN in the anterior prefrontal cortex and dPCC (Fig 3B; Table 2), which are nodes associated with the DMN, once again suggesting increased connectivity between the DMN and TPN.

**Table 2:** Voxel wise Group Differences.

	Region	Abbreviation	Coordinates	Voxels
DMN				
**Chronic mTBI > Control**	Dorsal anterior cingulate/premotor	dACC/PM	(-4, 22, 34)	1034
	Right Insula	R In	(46, 4, 2)	802
	Left Insula	L In	(-40, 10, 6)	698
	Left dorsolateral prefrontal cortex	L DLPFC	(-32, 46, 36)	414
**Chronic mTBI < Control**	Dorsal posterior cingulate cortex	dPCC	(8, -52, 38)	415
	Left supramarginal gyrus	L SMG	(-36, -64, 52)	791
**TPN**				
**Chronic mTBI > Control**	Anterior prefrontal cortex	APFC	(-4, 42, 0)	559
	Dorsal posterior cingulate cortex	dPCC	(10, -36, 36)	535
**Acute PCS > no PCS**	Right angular gyrus	R AnG	(30, -68, 4)	620

### Resting State fMRI-ROI Based Analysis

#### Cross Sectional Analysis

**Fig 4:**
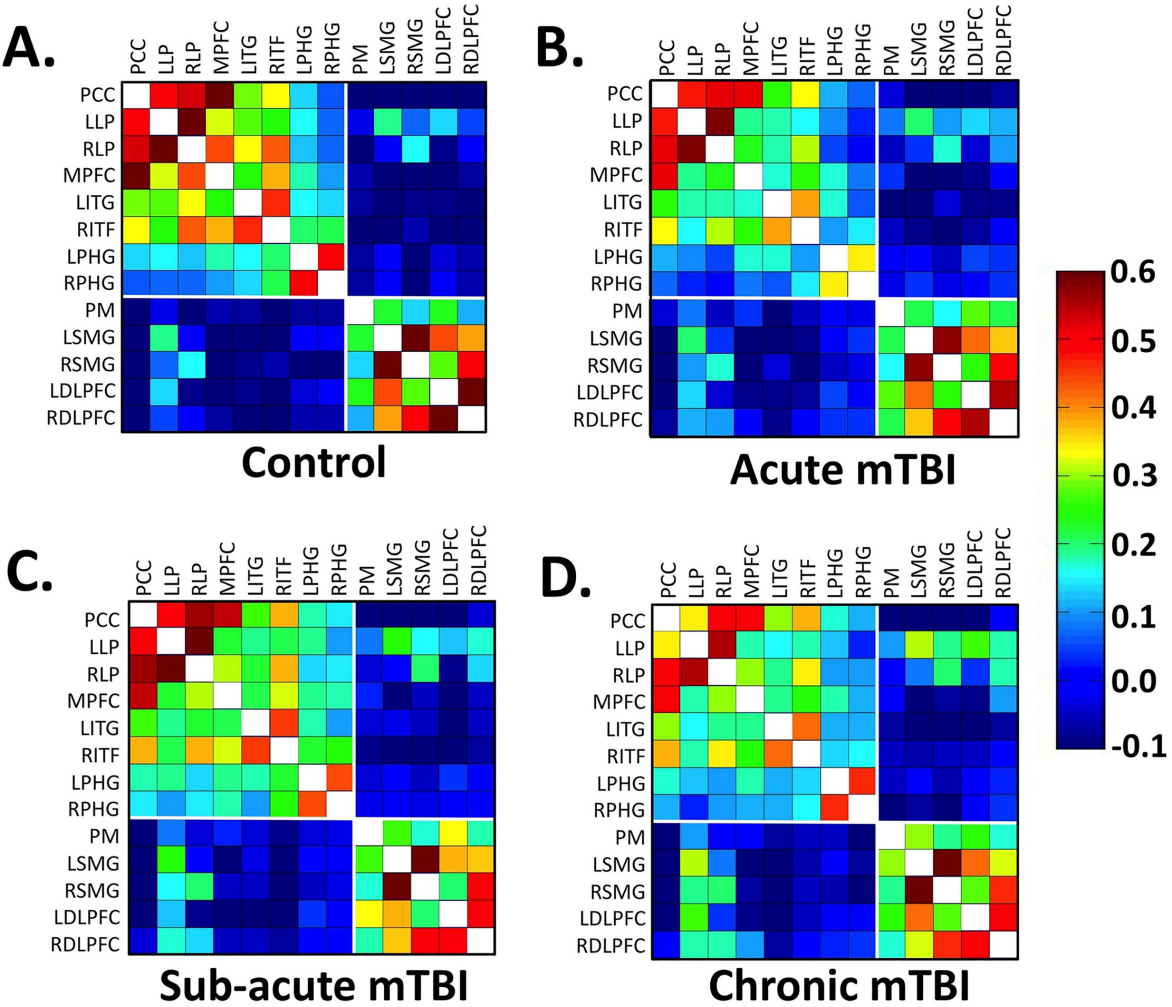
Average correlation matrices. Matrices illustrating both within and between network connectivity values for the DMN and TNP for (A) Controls (B) Acute mTBI (C) Sub-acute mTBI (D) Chronic mTBI. The white boundaries separate the Default Mode Network (DMN) and the Task Positive Network (TPN) regions of interest (from top of matrix to bottom of matrix respectively). The color bar represents z-scores.

**Fig 5:**
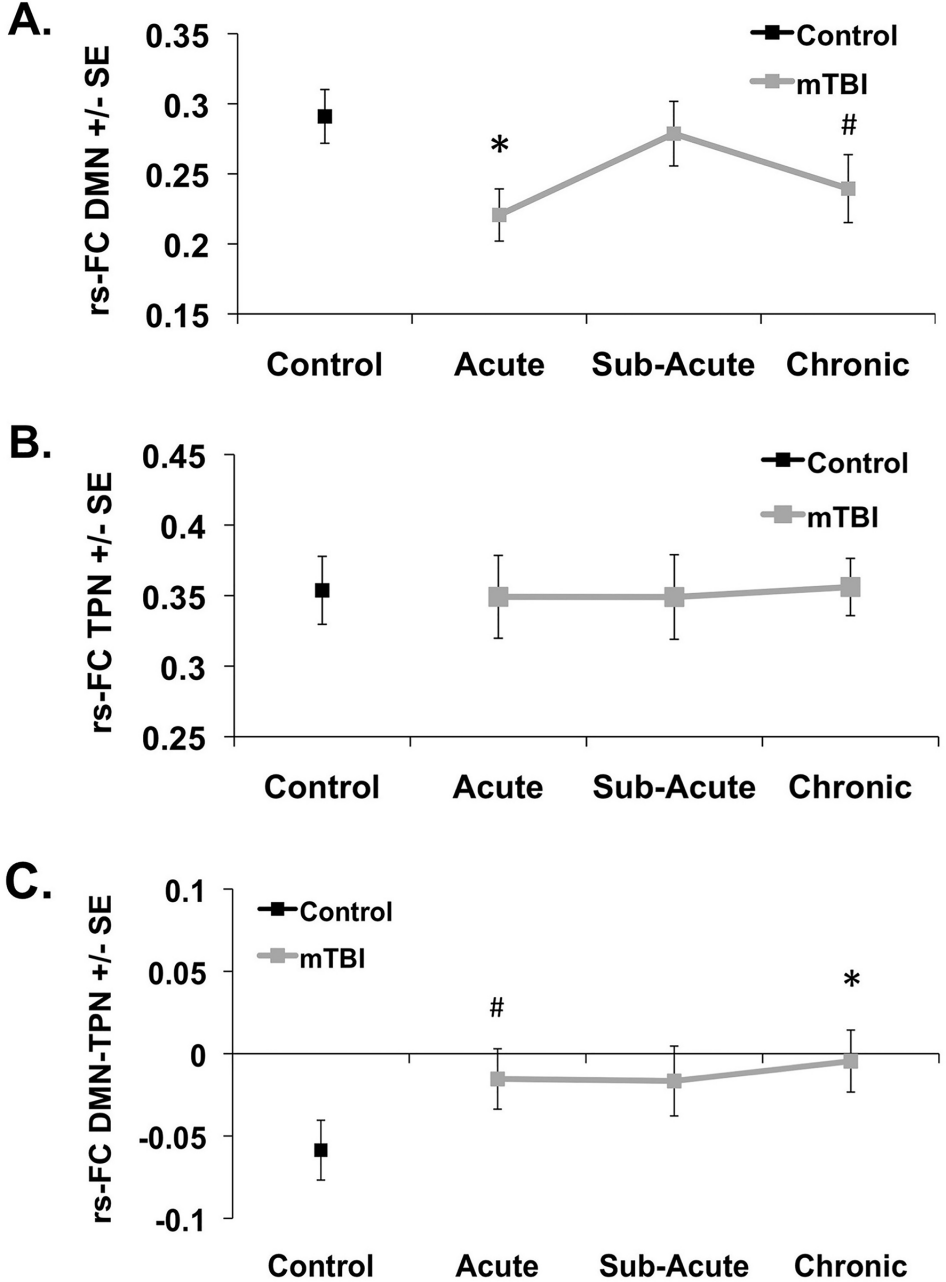
Plots of average network resting state connectivity strength. Plots of strength +/- standard error (SE) for control group and mTBI group across three time points. Average strength of network rs-FC for the (A) within Default Mode Network (rs-FC DMN) and (B) within Task Positive Network (TPN) (rs-FC TPN) and (C) between DMN and TPN (rs-FC DMN-TPN). # p <0.1, * p <0.05 compared to the control group based on analysis of covariance.

Average pairwise correlation matrices are shown for the control group (Fig 4A) and the mTBI group at each time point (Fig 4B–4D). These matrices visualize that for each group, the connectivity within each network (top left and bottom right corner of each matrix) is greater than connectivity between the two networks (top right and bottom left corner of each matrix). When the strength of rs-FC within the DMN was calculated, compared to the control group reduced DMN rs-FC was noted in the mTBI group in the acute stage (F = 9.088; p = 0.004) and a trend in reduced DMN rs-FC in the chronic stage (F = 3.027; p = 0.088). No differences in DMN rs-FC were noted in the mTBI group in the sub-acute stage (F = 0.185; p = 0.669) (Fig 5A). Compared to the control group, mTBI patients did not show altered strength of rs-FC within the TPN at any time point (all p-values > 0.500) (Fig 5B). Compared to the control group, mTBI patients showed increased between network connectivity as demonstrated by increased DMN-TPN rs-FC in the acute (F = 2.851; p = 0.097) and chronic (F = 4.200; p = 0.045) stages of injury although it did not reach statistical significance in the acute stage. No differences in DMN-TPN rs-FC were noted in the mTBI group in the sub-acute stage (F = 2.254; p = 0.139) (Fig 5C).

#### MTBI Longitudinal Analysis

Results of the repeated measures ANCOVAs in the mTBI group failed to detect longitudinal differences in DMN rs-FC (F = 0.443; p = 0.643), TPN rs-FC (F = 1.283; p = 0.282), or DMN-TPN rs-FC (F = 0.529; p = 0.591).

### Resting CBF Analysis

#### Cross Sectional Analysis

**Fig 6:**
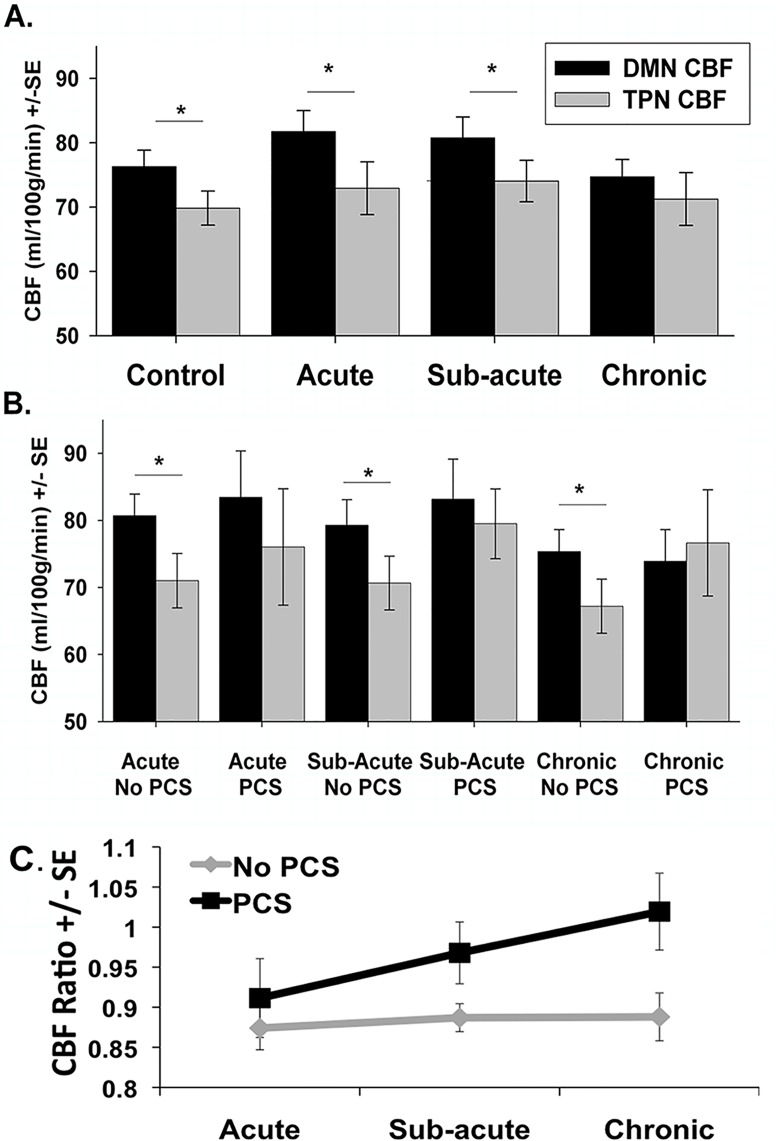
Plots of average network cerebral perfusion flow (CBF). Plots of average network CBF values for the Default Mode Network (DMN) shown in Black and the Task Positive Network (TPN) shown in gray. (A) Average network CBF values for control participants and mTBI group across the three time points. (B) Average network CBF values for post-concussive syndrome (PCS) group and non PCS mTBI group. (C) CBF ratio (TPN CBF/DMN CBF) for the PCS group and non PCS group. * p <0.05 based on paired t-tests.

Compared to the control group, there was no group differences in network CBF values for either the DMN CBF or TPN CBF for the mTBI group as a whole at any of the three time points (all p-values > 0.050). Furthermore, there were no differences in the CBF ratio between the mTBI group and the control group at any time point. As has been demonstrated by others [46], there was significantly greater DMN CBF than TPN CBF in the control group (p = 0.002). There was also greater DMN CBF compared to TPN CBF in the mTBI population as a whole at the acute stage (p <0.001), and in the sub-acute stage (p = 0.007) (Fig 6A). However, at the chronic stage, mTBI patients failed to maintain a significant difference between DMN CBF and TPN CBF (p = 0.452) (Fig 6A).

#### MTBI Longitudinal Analysis

Results of the repeated measures ANCOVAs in the mTBI group failed to detect longitudinal differences in DMN CBF, TPN CBF, or the CBF ratio.

#### Exploratory PCS Longitudinal Analysis

There was significantly greater DMN CBF compared to the TPN CBF in the mTBI group without PCS at all three time points (acute: p<0.001; sub-acute: p<0.001; chronic: p = 0.004). However, there were no significant differences between the TPN CBF and the DMN CBF in the mTBI group with PCS at any time point (acute: p = 0.106; sub-acute: p = 0.659; chronic: p = 0.195) (Fig 6B).

Based on the results of the repeated measures ANCOVA, there were no between group differences or within group differences for DMN CBF or TPN CBF. For the CBF ratio, there were no significant within group differences (F = 1.050; p = 0.358) or between group differences (F = 2.002; p = 0.171) (Fig 6C).

## Discussion

Given the association between rs-FC between the DMN and TPN and cognition in prior studies among both healthy participants and various patient populations [16–20], we predicted that rs-fMRI and perfusion ASL may be a sensitive measure to help distinguish mTBI patients from a control group. Investigating both perfusion ASL and rs-fMRI across three time points in a mTBI population, we demonstrated for the first time both a shift in network functioning common to all mTBI patients regardless of persistent symptoms, as well as preliminary results suggesting an adjustment in network perfusion within the mTBI population specific to patients with chronic PCS. The findings of this study lead us to three core conclusions. First, while the results failed to detect longitudinal changes in resting state measures within the mTBI population, the results do show that mTBI patients demonstrate increased rs-FC between the DMN and TPN at the chronic stage but reduced strength of rs-FC within the DMN at the acute stage. Second, chronic mTBI patients fail to maintain the expected balance of resting state perfusion between the DMN and TPN compared to the control population. Finally, results from the exploratory analysis suggest that mTBI patients who develop chronic PCS demonstrate modified network perfusion patterns across all three stages of injury when compared to those who do not develop persistent symptoms.

### Cross Sectional rs-FC

While mTBI patients performed similarly to control participants on all of the neuropsychological assessments, alterations in rs-FC and perfusion pertaining to the nodes of these two networks were noted mainly during the chronic stage (Fig 3). As previously shown by other groups [34,59], mTBI patients demonstrated a trend in reduced rs-FC within the DMN at the chronic stage (Fig 5A). Furthermore, similar to previous studies that have demonstrated significant reduction in rs-FC in the TBI population shortly after injury [30,33], the mTBI patients in this study demonstrated a reduction in DMN rs-FC during the acute stage of injury. However, the mTBI patients in this study failed to demonstrate reductions in DMN rs-FC during the sub-acute stage. This inconsistent findings across studies may be due to differences in time of observation since injury, the inclusion criteria chosen by the various studies as some studies only included mTBI patients with cognitive complaints or post-traumatic symptoms, as well as the variability in the rate of recovery of patients within the first month following mTBI [34]. Furthermore, this variability may be due to the limited sensitivity of the current techniques to measure the subtle injury induced my mTBI. On the other hand, the mTBI group in this study demonstrated increased rs-FC between the DMN and TPN at the chronic stage at the ROI level (Fig 5C). Furthermore, based on the voxel-wise analysis, there is increased rs-FC between the DMN and the L DLPFC node of the TPN as well as between the TPN and the MPFC and dPCC nodes of the DMN (Fig 3).

It is interesting to note that similar to our previous report on sub-acute mTBI patients with memory complaints [38], the chronic mTBI patients demonstrated increased rs-FC between the DMN and the dorsal ACC and bilateral insula (Fig 3A). While the bilateral insula is sometimes coactivated with the TPN (Fig 2B) [12], the insula has another role as part of the SN [23]. The main role of the SN is to detect salient sensory stimuli and modulate mental transitions between the TPN and DMN, directing focus internally or externally based on this detected stimuli [23,25]. Using Granger causality, it has been suggested that the activity in the right anterior insula node of the SN precedes activity in the TPN and DMN supporting the notion that the SN modulates the activations and deactivations of the DMN and TPN [24]. The increased rs-FC between the DMN and regions associated with the SN in our patient population suggests an increased need of the SN to modulate activity of a disrupted DMN, leading us to postulate that this may be one of the causes for increased cognitive fatigue in these patients [60].

### Cross Sectional ASL Perfusion

In addition to alterations in rs-FC, an imbalance between resting perfusion values within the DMN and TPN was also noted in the chronic stage. The fact that such changes appear at the chronic stage suggests that recovery from mTBI is a constantly evolving mechanism that progresses over time possibly to help balance the basal activity of these two networks. Further, it could be hypothesized that this increased allocation of CBF to the TPN compared to the DMN may contribute the common symptoms of increased cognitive fatigue [60] or attentional deficits [61] typically noted in mTBI patients. In other words, mTBI patients may have a reduced ability to maintain normative resting conditions. This may also explain previous reports of altered activation and deactivation patterns noted in task based fMRI studies [22,59,62]. Furthermore, Bonnelle et al., reported an association between deactivation of the DMN and structural integrity of the fiber tract connecting the nodes of the right anterior insula and pre-supplemental motor area (nodes of the SN) [62]. While this previous finding lends further support to the notion that the perfusion imbalance between the DMN and TPN noted in the present study may be due to subtle shearing injuries caused by mTBI, this current analysis does not provide evidence to support this notion. While our study lacks task based functional data or diffusion tensor imaging (DTI) data, our findings suggest an intriguing imbalance in the communication between the DMN and TPN at rest. However, to fully understand these disparities, further research similar to that of Bonnelle and colleagues (2012) is needed in task induced activation and deactivation patterns in mTBI patients as well as further investigation into linking structural damage as measured by DTI with altered functional communication. However, taken together, the shift in the balance between the DMN, TPN, and SN in both rs-FC and resting perfusion ASL provides evidence for the Default Mode Interference Hypothesis in mTBI [14]. The imbalance between these three networks at rest evolves over the course of recovery possibly contributing to the cognitive deficits and persistent symptoms experienced by a significant subset of mTBI patients.

### Longitudinal ASL Analysis

While the results do not show longitudinal changes in resting state or perfusion measures for the mTBI group as a whole, to further assess the changes in regional CBF, the mTBI group was further divided into those with and without chronic PCS (defined here as those still experiencing significant symptoms at least 6 months following injury.) While the primary goal was to see how the disruptions between the DMN and TPN affected patient symptoms and cognitive performance, a secondary goal was to investigate alterations in these networks in the acute stage in patients with and without persistent symptoms.

Similar to our findings in the chronic mTBI population as a whole, the mTBI patients experiencing chronic PCS failed to maintain the expected balance between perfusion to the DMN and the TPN (Fig 6B). The TPN nodes show greater perfusion compared to the DMN in the chronic stage in patients experiencing chronic PCS. Furthermore, this alteration in resting perfusion appears in mTBI patients with PCS at all three stages of injury, while those without PCS maintain a similar pattern of network perfusion as the control participants. This finding suggests that there may be a unique pattern of network perfusion in mTBI patients in the initial stages of injury, which may be predictive of whom may develop chronic PCS. However, given the difference in age between these two mTBI sub-groups, it is plausible that age has an influence on alterations in networks perfusion following injury. It has been previously reported that older TBI patients are more likely to develop persistent symptoms [63] and our data may suggest that the altered CBF ratio may contribute to this increased likelihood of PCS among older patients. Since this trend in network perfusion is seen across all stages of injury in mTBI patients with chronic PCS, further research is necessary to test and validate such CBF changes as a possible marker for predicting long-term outcome of mTBI patients. Future studies with larger sample size should be designed to separate the effect of age to understand pure mTBI related changes.

### Limitations and Future Directions

The findings of a modified interaction between the TPN and DMN among mTBI patients and especially among mTBI patients with chronic PCS should be taken in the context of the limitations of the study. The control group used in this study included healthy normal controls and not patients who suffered non-CNS related injury. It is possible that our choice of control participants could undermine some of the predisposing factors in the mTBI group. While our control participants were only scanned at one time point, recent groups have reported on the reliability of resting state measures within control populations [64,65], allowing us to be confident that the changes noted on the mTBI population are not due to variability within the technique. In addition, it is possible that the results presented from the ROI analysis in this manuscript may have been potentially biased by the method of ROI selection. The ROIs were selected from the results from the voxel-wise analysis of an independent control group [57]. However, future work using more data driven approaches such as independent component analysis (ICA) is warranted to further validate our findings. Furthermore, the screening included in this study did not account for effort or litigation status. However, the main findings presented in this analysis are collected during resting state conditions, which should not be affected by differences in effort or malingering. Finally, the association between a static perfusion measure of CBF and altered synchronization of the BOLD signals across different regions is an area of active investigation in the field. With multiple groups investigating resting state functional connectivity based off of dynamic CBF measures [66,67] future work probing CBF based functional connectivity within the TBI population is needed to provide a more complete understanding of functional damage induced by this injury. However, in spite of the aforementioned limitations, to our knowledge, this is the first longitudinal analysis across three time points that links the changes in resting state connectivity and an underlying altered resting state perfusion within these networks across multiple stages of injury.

## Conclusion

In conclusion, our data indicates an inability of mTBI patients to modulate the balance between the DMN and TPN at rest, as evidenced by alterations in network interactions in rs-FC and network resting perfusion measures across the first 6 months following injury. Our results are novel in that they extend the notion of DMN Interference in a mTBI population to include not only increased rs-FC between the networks, but also alterations in resting network perfusion. While these alterations are present in mTBI patients in the chronic stages, alterations in perfusion are present earlier in the acute and sub-acute stages in mTBI patients who suffer from persistent symptoms suggestive of distinctive courses of functional recovery in those with and without persistent symptoms. These findings raise the possibility that these resting state measures and altered perfusion may provide additional insight and aid clinicians in predicting the long-term outcomes of patients with mTBI.

## Supporting Information

S1 Table Individual Demographic and Clinical Characteristics. (DOCX)

S2 Table MNI coordinates for the Default Mode Network (DMN) and Task Positive Network (TPN) regions. (DOCX)

S3 Table DMN Clusters for each group. (DOCX)

S4 Table TPN Clusters for each group. (DOCX)
