# Supplementary material for: Disruptions in Resting State Functional Connectivity and Cerebral Blood Flow in Mild Traumatic Brain Injury Patients
Source: PLoS One. 2015 Aug 4;10(8):e0134019. doi: 10.1371/journal.pone.0134019 (PMC4524606; doi:10.1371/journal.pone.0134019)
Supplement: S1 Table — (DOCX) [file pone.0134019.s001.docx]

**Supplemental Table 1: Individual Demographic and Clinical Characteristics**

| **GCS** | **GOSE** | **Age** | **Sex**  **(M:1 F:0)** | **Education** | **Days Acute** | **Days**  **Sub-acute** | **Days Chronic** | **PCS** | **Mechanism** | **+CT** | **+MR** |
| --- | --- | --- | --- | --- | --- | --- | --- | --- | --- | --- | --- |
| 15 | 8 | 41 | 1 | 18 | 9 | 58 | 190 | 0 | MVC | 0 | 0 |
| 15 | 6 | 59 | 1 | 18 | 6 | 33 | 215 | 0 | Fall | 0 | 0 |
| 15 | 8 | 53 | 1 | 20 | 6 | 34 | 214 | 0 | Bicycle | L frontal lobe cortical contusion | |
| 14 | 8 | 20 | 1 | 14 | 10 | 32 | 158 | 0 | Assault | 0 | 0 |
| 15 | 8 | 18 | 1 | 10 | 5 | 32 | 163 | 0 | Sports | 0 | 0 |
| 15 | 8 | 24 | 1 | 16 | 4 | 27 | 266 | 0 | MVC | 0 | 0 |
| 15 | 8 | 26 | 1 | 14 | 2 | 38 | 148 | 0 | MVC | 0 | 0 |
| 15 | 8 | 27 | 1 | 16 | 7 | 28 | 199 | 0 | Sports | 0 | 0 |
| 15 | 8 | 19 | 1 | 10 | 1 | 33 | 203 | 0 | Fall | 0 | 0 |
| 15 | 6 | 28 | 0 | 12 | 7 | 24 | 216 | 0 | Fall | L frontal lobe punctate parenchymal contusion | |
| 15 | 8 | 65 | 0 | 14 | 10 | 88 | 223 | 0 | Fall | 0 | 0 |
| 15 | 8 | 30 | 0 | 16 | 6 | 30 | 215 | 0 | Bicycle | 0 | 0 |
| 15 | 8 | 18 | 1 | 12 | 9 | 28 | 197 | 0 | Fall | 0 | 0 |
| 15 | 8 | 49 | 1 | 10 | 1 | 36 | 178 | 0 | Assault | 0 | 0 |
| 15 | 5 | 29 | 1 | 16 | 10 | 28 | 211 | 0 | Assault | 0 | 0 |
| 15 | 8 | 26 | 0 | 13 | 7 | 39 | 194 | 0 | Fall | R SAH |  |
| 15 | 6 | 23 | 0 | 12 | 4 | 48 | 218 | 1 | MVC | 0 | 0 |
| 14 | 8 | 59 | 0 | 18 | 10 | 39 | 200 | 1 | Fall | 0 | 0 |
| 15 | 8 | 38 | 1 | 12 | 1 | 38 | 202 | 1 | MVC | 0 | 0 |
| 15 | 8 | 53 | 1 | 14 | 3 | 39 | 177 | 1 | MVC | 0 | 0 |
| 15 | 8 | 66 | 0 | 14 | 9 | 37 | 218 | 1 | MVC | 0 | 0 |
| 15 | 8 | 47 | 1 | 10 | 11 | 51 | 218 | 1 | Assault | Epidural hematoma and L occipital lobe hemorrhagic contusion | |
| 15 | 8 | 40 | 0 | 12 | 7 | 25 | 137 | 1 | Fall | 0 | 0 |
| 15 | 7 | 56 | 1 | 16 | 10 | 31 | 214 | 1 | Bicycle | L frontal lobe brain contusions and R frontal lobe focal SAH | |
| 14 | 5 | 38 | 1 | 13 | 3 | 24 | 204 | 1 | MVC | 0 | Bilateral frontal and R temporal contusions |
| 13 | 5 | 60 | 0 | 12 | 10 | 27 | 188 | 1 | MVC | 0 | 0 |
| 15 | 6 | 52 | 1 | 14 | 8 | 36 | 188 | 1 | Fall | 0 | 0 |
| 15 | 8 | 23 | 0 | 12 | 9 | 33 | 189 | 1 | MVC | 0 | 0 |

***SAH: subarachnoid hemorrhage**
