# Supplementary material for: Disruptions in Resting State Functional Connectivity and Cerebral Blood Flow in Mild Traumatic Brain Injury Patients
Source: PLoS One. 2015 Aug 4;10(8):e0134019. doi: 10.1371/journal.pone.0134019 (PMC4524606; doi:10.1371/journal.pone.0134019)
Supplement: S2 Table — (DOCX) [file pone.0134019.s002.docx]

**Supplemental Table 2: MNI coordinates for the Default Mode Network (DMN) and Task Positive Network (TPN) regions**

| **ROI Legend** | |
| --- | --- |
| **Region** | **MNI Coordinates (x, y, z)** |
| **DMN** | |
| RLP (Right lateral parietal region) | (46, -62, 36) |
| PCC (Posterior cingulate cortex) | (4, -48, 24) |
| LLP (Left lateral parietal region) | (-36, -66, -36) |
| MPFC (Medial prefrontal cortex) | (6, 50, -4) |
| RITG (Right inferior temporal gyrus) | (62, -6, -22) |
| RMTL (Right medial temporal lobe) | (30, -36, -16) |
| LMTL (Left medial temporal lobe) | (-26, -36, -18) |
| LITG (Left inferior temporal gyrus) | (-56, -8, -26) |
| **TPN** | |
| LDLPFC (Left dorsolateral prefrontal cortex) | (-42, 32, 12) |
| RDLPFC (Right dorsolateral prefrontal cortex) | (44, 36, 12) |
| LSMG (left supramarginal gyrus) | (-42, -42 42) |
| RSMG (right supramarginal gyrus) | (50, -42, 50) |
| PM (Premotor Ara) | (-6, 16,46) |
