# Supplementary material for: Disruptions in Resting State Functional Connectivity and Cerebral Blood Flow in Mild Traumatic Brain Injury Patients
Source: PLoS One. 2015 Aug 4;10(8):e0134019. doi: 10.1371/journal.pone.0134019 (PMC4524606; doi:10.1371/journal.pone.0134019)
Supplement: S3 Table — (DOCX) [file pone.0134019.s003.docx]

**Supplemental Table 3: DMN Clusters for each group**

|  | k | x | y | z |
| --- | --- | --- | --- | --- |
| Control | | | | |
|  | 22066 | -30 | 22 | 58 |
|  | 13015 | -2 | -4 | 26 |
|  | 3178 | 18 | -88 | -38 |
|  | 2847 | 44 | -66 | 48 |
|  | 2562 | 64 | -8 | -20 |
|  | 392 | 8 | -50 | -46 |
|  | 287 | 30 | -20 | -22 |
| V1 | | | | |
|  | 14972 | -20 | 32 | 48 |
|  | 8671 | 4 | -48 | 30 |
|  | 3067 | -44 | -64 | 38 |
|  | 1994 | 54 | -64 | 28 |
|  | 966 | 44 | -76 | -40 |
|  | 905 | -58 | 0 | -34 |
|  | 738 | 56 | -4 | -34 |
|  | 360 | -42 | -78 | -44 |
|  | 301 | -4 | -56 | -46 |
| V2 | | | | |
|  | 16523 | -6 | 54 | 6 |
|  | 8503 | -6 | -56 | 22 |
|  | 5263 | -46 | -64 | 30 |
|  | 2300 | 54 | -68 | 30 |
|  | 1980 | 52 | 2 | -44 |
|  | 558 | -26 | -32 | -20 |
|  | 453 | 26 | -28 | -22 |
|  | 398 | 12 | -88 | -38 |
|  | 374 | 46 | -64 | -44 |
| V3 | | | | |
|  | 19002 | 10 | 48 | 4 |
|  | 8984 | 0 | -46 | 30 |
|  | 4270 | 50 | -62 | 28 |
|  | 2951 | -50 | -72 | 36 |
|  | 1941 | -62 | -26 | -20 |
|  | 540 | -22 | -24 | -24 |
|  | 534 | -30 | 20 | -24 |
|  | 367 | 36 | 34 | -16 |
|  | 366 | 36 | -72 | -36 |
