# Supplementary material for: Disruptions in Resting State Functional Connectivity and Cerebral Blood Flow in Mild Traumatic Brain Injury Patients
Source: PLoS One. 2015 Aug 4;10(8):e0134019. doi: 10.1371/journal.pone.0134019 (PMC4524606; doi:10.1371/journal.pone.0134019)
Supplement: S4 Table — (DOCX) [file pone.0134019.s004.docx]

**Supplemental Table 4: TPN Clusters for each group.**

|  | k | x | y | z |
| --- | --- | --- | --- | --- |
| Control | | | | |
|  | 1002 | 44 | 36 | 12 |
|  | 7898 | -44 | 32 | 12 |
|  | 5386 | -42 | -42 | 42 |
|  | 5189 | 50 | -42 | 56 |
|  | 2645 | -52 | -50 | -20 |
|  | 1807 | 10 | -78 | -32 |
|  | 1295 | 56 | -60 | -16 |
|  | 1240 | 2 | 28 | 48 |
|  | 349 | -12 | 6 | 8 |
|  | 318 | -2 | 8 | 28 |
| Acute | | | | |
|  | 10719 | -44 | -44 | 44 |
|  | 94595 | 46 | 36 | 16 |
|  | 6708 | -44 | 36 | 14 |
|  | 1504 | 4 | 4 | 28 |
|  | 1091 | -54 | -62 | -16 |
|  | 970 | -26 | 8 | 60 |
|  | 863 | 54 | -46 | -20 |
|  | 570 | 10 | -38 | -42 |
|  | 310 | -26 | 36 | -20 |
|  | 266 | 8 | 6 | 2 |
| Sub-acute | | | | |
|  | 9417 | 48 | 38 | 12 |
|  | 8537 | -40 | 34 | 12 |
|  | 4411 | -58 | -36 | 42 |
|  | 4181 | 50 | -42 | 54 |
|  | 2404 | -2 | 8 | 28 |
|  | 1561 | -58 | -58 | -16 |
|  | 1258 | 60 | -50 | -14 |
|  | 977 | 8 | -38 | 44 |
|  | 330 | -46 | -22 | 4 |
|  | 295 | -16 | -2 | 18 |
|  | 275 | 20 | -78 | -50 |
| Chronic | | | | |
|  | 14505 | 46 | 42 | 8 |
|  | 13764 | -38 | -54 | 54 |
|  | 10605 | -40 | 34 | 12 |
|  | 1213 | -56 | -56 | -20 |
|  | 1054 | 58 | -44 | -22 |
|  | 930 | 8 | -36 | 38 |
|  | 338 | -20 | -98 | 14 |
|  | 8984 | 0 | -46 | 30 |
|  | 4270 | 50 | -62 | 28 |
|  | 2951 | -50 | -72 | 36 |
|  | 1941 | -62 | -26 | -20 |
|  | 540 | -22 | -24 | -24 |
|  | 534 | -30 | 20 | -24 |
|  | 367 | 36 | 34 | -16 |
|  | 366 | 36 | -72 | -36 |
